# Chiropractic Spinal Manipulation and Fall Risk in Older Adults With Spinal Pain: Observational Findings From a Matched Retrospective Cohort Study

**DOI:** 10.7759/cureus.72330

**Published:** 2024-10-24

**Authors:** Robert J Trager, Wren M Burton, Julia V Loewenthal, Jaime A Perez, Anthony J Lisi, Matthew H Kowalski, Peter M Wayne

**Affiliations:** 1 Department of Chiropractic Medicine, Connor Whole Health, University Hospitals Cleveland Medical Center, Cleveland, USA; 2 Department of Family Medicine and Community Health, Case Western Reserve University School of Medicine, Cleveland, USA; 3 Department of Biostatistics and Bioinformatics Clinical Research Training Program, Duke University School of Medicine, Durham, USA; 4 Osher Center for Integrative Health, Harvard Medical School, Brigham and Women’s Hospital, Boston, USA; 5 Division of Preventive Medicine, Harvard Medical School, Brigham and Women’s Hospital, Boston, USA; 6 Division of Aging, Harvard Medical School, Brigham and Women’s Hospital, Boston, USA; 7 Clinical Research Center, University Hospitals Cleveland Medical Center, Cleveland, USA; 8 Pain Research, Informatics, Multimorbidities, and Education (PRIME) Center, Veterans Affairs (VA) Connecticut Healthcare System, West Haven, USA; 9 Section of Biomedical Informatics and Data Science, School of Medicine, Yale University, New Haven, USA

**Keywords:** accidental falls, aged, back pain, chiropractic, cohort studies, geriatrics, spinal manipulation

## Abstract

Introduction: Limited research suggests that spinal manipulative therapy (SMT) might positively influence balance, yet its association with falls remains underexplored. We hypothesized that older adults receiving chiropractic SMT for spinal pain would have a reduced fall risk during 13 months of follow-up compared to matched controls.

Methods: We searched >116 million patient records from TriNetX (2013-2023; Cambridge, MA, US) to identify adults aged ≥65 years with spinal pain. After excluding those with major fall risk factors, we formed SMT and non-SMT cohorts, using propensity score matching to control for confounders (e.g., age, sex, comorbidities). Risk ratios (RR) with 95% CIs and p-values were calculated for primary (fall) and secondary (limb fracture) outcomes over 13 months. We explored the cumulative incidence of falls and fractures and negative control outcomes (colonoscopy, vital signs, diabetes, nicotine/tobacco screening).

Results: After matching, each cohort had 1,666 patients (mean age 72 years). The SMT cohort had a lower fall rate than the non-SMT cohort (3.8% vs. 5.4%), yielding an RR (95% CI) of 0.71 ((0.52, 0.97); p=0.0319). Cumulative incidences revealed a brief lag in SMT cohort fall incidence. There was no meaningful difference in limb fractures (RR of 1.16 (0.87, 1.54); p=0.3153). Negative control outcomes were similar between cohorts.

Conclusions: This study suggests that older adults receiving SMT for spinal pain may have a reduced risk of falls. However, given the observational nature of the study and the lack of significant differences in limb fracture incidence, the clinical significance of these findings remains uncertain. Further research, including randomized controlled trials, is needed to explore injurious falls, care utilization, pain, and costs.

## Introduction

Falls are a major concern for the growing global aging population [1]. They can lead to adverse outcomes, including fractures and hospitalizations [2], and are the leading cause of fatal and nonfatal injuries among adults age 65 and older in the United States (US), the setting of the current study [3]. Chiropractors are clinicians who commonly use spinal manipulative therapy (SMT) to manage spinal pain disorders affecting older individuals [4]. While some evidence suggests SMT improves mobility and measures of balance, limited research has explored whether this therapy influences the likelihood of falling [5,6].

According to US data from 2018, 27.5% of older adults reported falling at least once in the past year [7]. Pain has been identified as an independent risk factor for falls in older individuals [8-10]. Spinal pain disorders may predispose older individuals to falls via impairments of balance, gait, and sensorimotor function [11-13].

Studies have found that SMT improves markers of sensorimotor function and multisensory integration in older adults [14], alleviates neck pain [15] and low back pain [16], and may improve cervicogenic dizziness [17]; however, there is limited evidence linking it to a reduction in the likelihood of falls [5]. Previous investigations mainly consisted of pilot and feasibility studies [18,19] and have focused on balance markers rather than fall occurrences [20], thereby necessitating additional research on this topic.

Research aim

This study aimed to investigate whether older adults (aged ≥65) with spinal pain who receive SMT have a reduced risk of falling over a 13-month follow-up window compared to matched controls who do not receive SMT. Specifically, we hypothesized that SMT would be associated with a significant decrease in the risk ratio (RR) of falls compared to a general medical examination alone.

## Materials and methods

Study design

This retrospective study included two cohorts, spanning a 10-year data range with patient inclusion ending 13 months prior to the query date (December 17, 2023). Reporting adheres to the Strengthening the Reporting of Observational Studies in Epidemiology (STROBE) statement. The University Hospitals Institutional Review Board (Cleveland, OH, US) determined this study represented "not human subjects research" (STUDY20230244), thus not requiring patient consent. Study methods followed a registered protocol [21].

Setting and data source

This study leveraged the US TriNetX network (Cambridge, MA, US), which includes de-identified, aggregated data from electronic medical records of 82 healthcare organizations and 116 million patients. Contributing organizations remain anonymous yet are typically academically affiliated. Standardized nomenclatures (e.g., International Classification of Disease 10th Edition (ICD-10) codes) can be used to query the dataset. ICD-9 coding is automatically interconverted to ICD-10. Markers of data completeness are available per query. One study estimated an 87% completeness for medication data within TriNetX [22].

To improve fall identification, we incorporated natural language processing [23,24]. TriNetX uses machine learning technology (Averbis, Freiburg im Breisgau, DE) to search text from clinical charts. This software recognizes the presence/absence of conditions based on context. Averbis has been validated with a Kappa value of 0.79 (good agreement) compared to manual chart review [25]. During pilot testing of Averbis via cross-sectional query in October 2023, we identified eight percent greater falls in the TriNetX dataset compared to ICD-10 codes alone.

The present study relied on real-world data derived from routine care. Measurement of variables such as visual disturbances, gait abnormalities, fear of falling, care provider dependency, and conductive and sensorineural hearing loss was based on diagnoses and free-text documentation recorded by healthcare professionals within the electronic health records. These conditions were captured through standardized coding systems and natural language processing, as described above.

Although specific characteristics of treating chiropractors in this study are obfuscated by TriNetX, chiropractors included in the network are employed by academic integrated healthcare organizations [26]. Chiropractors in these settings are typically affiliated with physical medicine and rehabilitation, physical therapy, or primary care departments and have several years of clinical experience [27]. These organizations are encouraged to follow Medicare’s meaningful use requirements for fall screening assessment among adults aged ≥65 [28].

Participants

Inclusion Criteria

Patients at least 65 years of age were included. To improve and standardize data completeness and healthcare utilization, we required a general medical examination or non-chiropractic visit type (i.e., inpatient, emergency, observation, pre-admission, or short-stay) within the 13 months preceding and following the index date of inclusion. Given that patients may only visit a primary care physician annually, an additional one-month buffer provided flexibility for inclusion, accommodating variations in the timing of prior routine visits.

To identify spinal pain disorders, we used the ICD-10 categories M50-54, which encompass degenerative and musculoskeletal spinal pain etiologies (Appendix 1). We focused on spinal pain, considering it increases fall risk and is often managed by chiropractors [4].

Patients in the SMT cohort were identified at the first co-occurrence of spinal pain and any Current Procedural Terminology code specifying chiropractic SMT (i.e., 98940, 98941, or 98942). Patients in the non-SMT cohort were identified at the first co-occurrence of spinal pain and general medical examination (Appendix 2).

Exclusion Criteria

Both cohorts were fall-naïve (i.e., no fall ≤ 91 days prior to the index date of inclusion), thereby improving comparability by excluding patients at risk of recurrent falls. Furthermore, this step helped align our methods with a new-user design, a feature encouraged in retrospective cohort studies [29]. Other exclusions encompassed individuals using a wheelchair, with dementia, and those undergoing surgery, bed-bound, visiting the emergency department, or receiving inpatient or critical care services on the index date. We excluded patients receiving SMT or similar manual therapies from the non-SMT cohort (Appendix 3).

Variables

We used the TriNetX platform’s online query builder feature to define our study cohorts. This tool allows researchers to enter eligibility criteria (see Participants and Appendices 1, 2, and 3), after which the platform retrieves and counts the patients who meet these criteria, making them available for further analysis [26,30]. Similarly, the TriNetX online analytics platform enables users to define matching parameters and enter outcomes based on standardized nomenclature and natural language processing terms [26,30].

Falls were identified using the ICD-10 subcategory W00-W19 codes, which describe accidental falls (slipping, tripping, stumbling, and falls) [31]. This subcategory includes falls caused by routine mechanisms such as ice and snow, falls from a bed, chair, stairs, or ladder, and falls due to bumping against objects or stepping into holes. However, it excludes falls resulting from assault, intentional self-harm, transport accidents, or falls from animals, machinery, or burning buildings. While W00-W19 codes are relatively accurate (i.e., 78% [32]), they may be underused. To address this, we employed natural language processing to improve fall ascertainment by searching free-text clinical notes for phrases related to the W00-W19 subcategory. This strategy enhances the code-based identification of falls. For the purposes of the present study, falls were treated as a dichotomous variable.

We assessed falls over a 13-month follow-up window, beginning the day after inclusion. While a one-year window is commonly used, we added a month to accommodate flexibility around the timing of annual medical examinations, during which fall screening often occurs.

We examined a secondary composite outcome of limb fractures of the shoulder, arm, forearm, wrist and hand, femur, leg, foot, and toe (ICD-10: S42, S52, S62, S72, S82, and S92). Limb fracture codes have a reported positive predictive value exceeding 70% for falls [33].

Statistical methods

Using the TriNetX online analytics platform, we implemented propensity score matching to balance confounding variables between cohorts, ascertaining data from two years preceding and including the index date. Propensity scores were calculated via Python’s scikit-learn package (version 3.7 Python Software Foundation, Delaware, USA) using logistic regression to estimate the log odds of non-SMT cohort assignment. Scores ranged from 0 (lowest likelihood) to 1 (highest). Patients were matched 1:1 using a greedy nearest-neighbor algorithm with a caliper of 0.1 pooled standard deviations of the logit of the propensity score based on covariates having an association with falls (Appendix 4) [34].

We compared variables via Pearson chi-squared tests and independent-sample t-tests. Standardized mean difference (SMD) was used to assess between-cohort balance, with a threshold of <0.1. The RR for falls was calculated by dividing the incidence proportion of falls in the SMT cohort by the non-SMT cohort, with significance evaluated at p<0.05. We did not make imputations for missing data. We used R (version 4.2.2, Vienna, AT) to calculate 95% confidence intervals (CIs) and ggplot2 to graph propensity score density, and as a sensitivity analysis, plot cumulative incidence of falls and limb fractures.

To further examine the success of propensity matching, we calculated RRs for binary negative control outcomes (i.e., those that should remain uninfluenced by SMT). These included a colonoscopy, diabetes screening, nicotine/tobacco screening, and vital signs measurement (i.e., respiratory rate, heart rate, body temperature, blood pressure, and weight or body mass index) during follow-up (Appendix 5). We targeted point RR estimates of 0.73-1.38, suggestive of a between-cohort balance.

We calculated a required sample size of 1,286 using G*Power (version 3.1.9.7, University of Kiel, DE) z-test to detect a between-cohort difference in fall incidence of five percent, with two tails, a power of 0.95, two-tailed α error of 0.05, and an allocation ratio of one.

## Results

Participants

We identified patients from several healthcare organizations (SMT: 11; usual care: 65). Before matching, there were 1,674 patients in the SMT cohort and 85,974 in the non-SMT cohort. After matching, each cohort had 1,666 patients (mean age 72 years). Before matching, patients in the SMT cohort were more likely to have disorders of the lens, choroid, retina, and spinal stenosis and were less likely to present for examinations or specific healthcare purposes, among other differences (SMD >0.1; Table 1). Following matching, all variables were optimally matched (SMD <0.1; Table 1), except for “fear of falling”; however, this variable had insufficient patients to enable statistical comparison.

**Table 1:** Baseline characteristics before and after propensity score matching SMD: standardized mean deviation, SMT: spinal manipulative therapy

Variable	Before matching			After matching		
	SMT	Non-SMT	SMD	SMT	Non-SMT	SMD
N	1,674	85,974		1,666	1,666	
Age	72.4 (5.5)	71.7 (5.9)	0.124	72.4 (5.5)	72.1 (5.7)	0.062
Female	967 (58%)	50560 (59%)	0.021	962 (58%)	965 (58%)	0.004
Male	707 (42%)	32151 (37%)	0.099	704 (42%)	700 (42%)	0.005
Diagnoses						
Abnormalities of gait and mobility	176 (11%)	11639 (14%)	0.093	172 (10%)	163 (10%)	0.018
Adverse socioeconomic/psychosocial circumstances	50 (3%)	5889 (7%)	0.179	50 (3%)	37 (2%)	0.049
Care provider dependency	35 (2%)	3442 (4%)	0.111	35 (2%)	23 (1%)	0.055
Conductive and sensorineural hearing loss	217 (13%)	5817 (7%)	0.209	213 (13%)	213 (13%)	<0.001
Disorders of bone density and structure	495 (30%)	24321 (28%)	0.028	490 (29%)	498 (30%)	0.011
Disorders of choroid and retina	295 (18%)	6163 (7%)	0.321	288 (17%)	261 (16%)	0.044
Disorders of lens	616 (37%)	13742 (16%)	0.486	608 (36%)	599 (36%)	0.011
Disorders of vestibular function	71 (4%)	2928 (3%)	0.044	70 (4%)	70 (4%)	<0.001
Dizziness and giddiness	378 (23%)	18323 (21%)	0.031	374 (22%)	357 (21%)	0.025
Encounters for other specific healthcare	235 (14%)	22512 (26%)	0.307	235 (14%)	209 (13%)	0.046
Fear of falling	0 (0%)	81 (0%)	0.043	0 (0%)	≤10 (1%)	0.110
Glaucoma	222 (13%)	6441 (7%)	0.190	221 (13%)	211 (13%)	0.018
Heart failure	232 (14%)	13151 (15%)	0.041	230 (14%)	208 (12%)	0.039
History of falling	47 (3%)	6520 (8%)	0.216	47 (3%)	45 (3%)	0.007
Hypotension	151 (9%)	7130 (8%)	0.026	146 (9%)	125 (8%)	0.046
Mood (affective) disorders	429 (26%)	22560 (26%)	0.014	425 (26%)	403 (24%)	0.031
Osteoarthritis	825 (49%)	37004 (43%)	0.125	821 (49%)	847 (51%)	0.031
Other and unspecified hearing loss	145 (9%)	9121 (11%)	0.066	145 (9%)	128 (8%)	0.037
Other diseases of the urinary system	406 (24%)	21257 (25%)	0.011	404 (24%)	395 (24%)	0.013
Other forms of heart disease	746 (45%)	36053 (42%)	0.053	740 (44%)	709 (43%)	0.038
Persons encountering health services for examinations	773 (46%)	82314 (96%)	1.303	773 (46%)	776 (47%)	0.004
Spinal stenosis	363 (22%)	11280 (13%)	0.227	356 (21%)	369 (22%)	0.019
Visual disturbances and blindness	210 (13%)	11511 (13%)	0.025	209 (13%)	185 (11%)	0.045
Procedures and medications						
Antidepressants	568 (34%)	29673 (35%)	0.012	564 (34%)	546 (33%)	0.023
Antihistamines	520 (31%)	32888 (38%)	0.152	519 (31%)	528 (32%)	0.012
Antipsychotics	135 (8%)	10767 (13%)	0.147	134 (8%)	126 (8%)	0.018
Beta blocking agents	725 (43%)	37854 (44%)	0.015	720 (43%)	709 (43%)	0.013
Gabapentinoids	440 (26%)	22239 (26%)	0.010	437 (26%)	432 (26%)	0.007
Gait training	128 (8%)	4931 (6%)	0.077	128 (8%)	112 (7%)	0.037
Intraocular lens procedures	98 (6%)	2005 (2%)	0.178	93 (6%)	82 (5%)	0.030
Loop diuretics	278 (17%)	17317 (20%)	0.091	276 (17%)	252 (15%)	0.039
Opioid analgesics	927 (55%)	54029 (63%)	0.152	922 (55%)	942 (57%)	0.024
Sedatives/hypnotics	667 (40%)	41888 (49%)	0.179	662 (40%)	660 (40%)	0.002

Data quality

The mean data points per patient per cohort were adequate (SMT: 6,660; usual care: 5,338). Following matching, the proportion of patients with an unknown age (SMT: 0%; non-SMT: 0%) was identical between cohorts, while the proportion having an unknown sex was similar, yet statistical comparison was precluded due to too few patients (SMT: 0%; non-SMT: ≤1%). After matching, cohorts’ propensity score densities overlapped, indicating adequate covariate balance (Appendix 6). The length of record was also adequate in both cohorts, with 99% and 98% of the SMT and non-SMT cohorts having at least two years of available data, respectively. These findings suggested minimal between-cohort differences regarding data density, completeness, and covariate balance.

Primary outcomes

The incidence of falls over 13 months following the index date was lower in the SMT cohort compared to the non-SMT cohort (Table 2). After propensity matching, 3.8% of the SMT cohort had a recorded fall, compared to 5.4% of the non-SMT cohort, yielding an RR (95% CI) of 0.71 ((0.52,0.97); p=0.0319).

**Table 2:** Likelihood of falls SMT: spinal manipulative therapy, RR: risk ratio, 95% CI: 95% confidence intervals, *: primary outcome

	Before matching		After matching	
Measure	SMT	Non-SMT	SMT	Non-SMT
N	1,674	85,974	1,666	1,666
Falls N (%)	64 (3.8%)	6,476 (7.5%)	64 (3.8%)	90 (5.4%)
RR (95% CI) for falls	0.51 (0.40,0.65; p<0.0001)	(reference)	0.71 (0.52,0.97; p=0.0319)*	(reference)

Secondary outcomes

**Figure 1:**
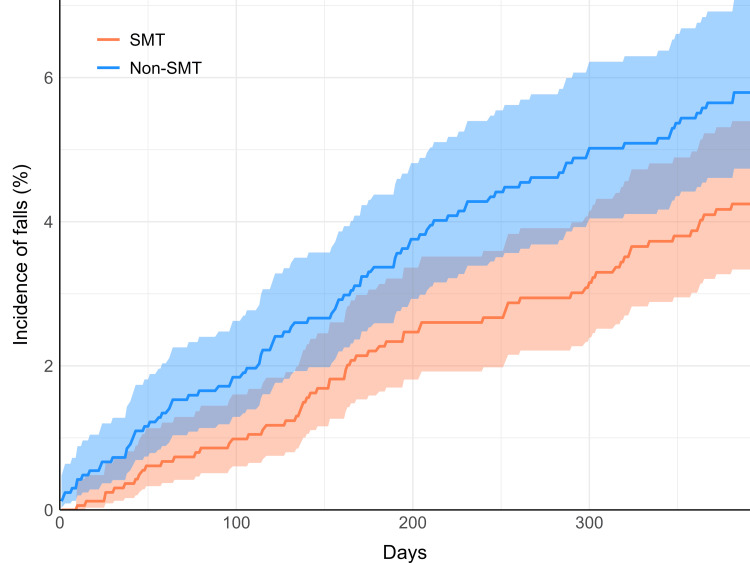
Cumulative incidence of falls Incidence curves in the SMT cohort (orange) and non-SMT cohort (blue) are shown over the 13-month follow-up period (395 days). Shaded regions indicate 95% CIs. SMT: spinal manipulative therapy, CIs: confidence intervals

A cumulative incidence plot (Figure 1) demonstrated that the incidence of falls increased linearly in both cohorts over the 13-month follow-up window. There was a brief initial period with few or no falls in the SMT cohort (approximately one to two weeks). Incidence curves did not intersect throughout. There was some expected overlap in 95% CIs, suggestive of imprecision on each day of follow-up. The overall risk of falls is best summarized by the RR, which accounts for the entire follow-up period (Table 1).

**Figure 2:**
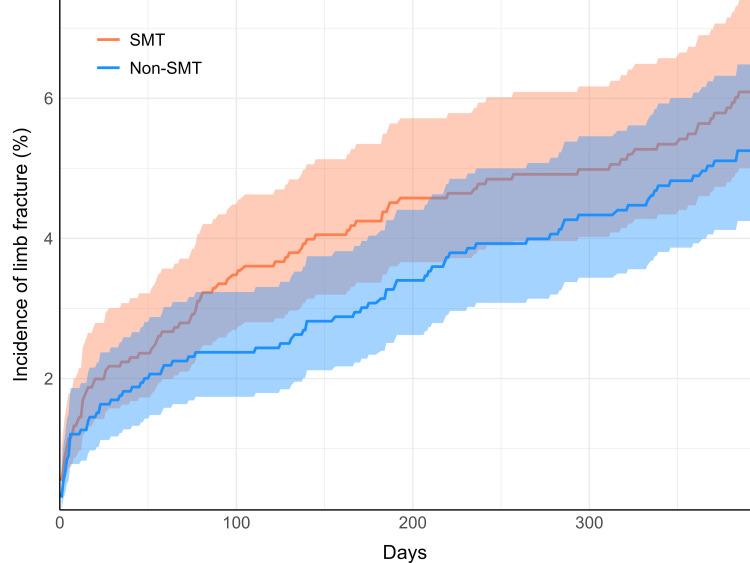
Cumulative incidence of limb fracture Incidence curves in the SMT cohort (orange) and non-SMT cohort (blue) are shown over the 13-month follow-up period (395 days). Shaded regions indicate 95% CIs. SMT: spinal manipulative therapy, CIs: confidence intervals

The incidence of limb fractures over 13 months following the index date of inclusion was not meaningfully different between cohorts. After matching, 5.7% (n=95) of the SMT cohort had a recorded limb fracture, compared to 4.9% (n=82) of the non-SMT cohort, yielding an RR (95% CI) of 1.16 ((0.87,1.54); p=0.3153). Cumulative incidences of fractures intersected within the first week and increased in a curvilinear manner (Figure 2).

The RRs for all negative control outcomes (95% CI) suggested no meaningful difference between cohorts (SMT vs. non-SMT): tobacco screening (0.99 (0.86, 1.15); p=0.8916), vital signs (0.90 (0.87, 0.93); p<0.0001), colonoscopy (1.22 (0.99, 1.50); p=0.0680), and diabetes screening (0.84 (0.81, 0.87); p<0.0001). These outcomes suggested similar healthcare engagement and other screening measures.

Regarding SMT utilization, 41% of patients in the SMT cohort had at least one additional SMT visit during follow-up. Among those with more than one SMT visit, the mean number of SMT follow-up visits was 7 (SD=6) while the median was 5, suggesting a positive skew with few patients having many follow-up SMT visits.

## Discussion

To our knowledge, this is the first adequately powered study to examine the association between SMT and falls. Our findings support our hypothesis that older adults with spinal pain receiving SMT have a reduced fall risk compared to matched controls not receiving SMT. This association persisted over 13 months despite only 41% of patients in the SMT cohort having more than one SMT visit. Similar negative control outcomes suggested that a reduced fall risk was not explained by differential healthcare engagement or screening. However, limb fracture risk was similar between cohorts, raising a question about the clinical significance of our findings.

A cumulative incidence graph demonstrated an initial lag in falls in the SMT cohort relative to the non-SMT cohort. While our time-to-event analysis was limited due to the de-identified nature of the data, this finding suggests SMT may have a stronger short-term protective effect against falls. Another potential explanation is the limited number of SMT visits, with over half of patients attending only a single session.

While survey data indicate a 27.5% annual fall incidence among older adults [7], we identified a lower incidence (i.e., non-SMT: 5.4% over 13 months). This discrepancy may stem from our exclusion of high-fall-risk individuals. In addition, survey methods may have a higher sensitivity to detect falls yet are subject to recall bias [7]. In contrast, a study that used an ICD-10-based query resembling ours reported a two-year fall incidence of 8.8%, translating to a low 4.4% annual rate [33]. Finally, our mean age (72 years) was relatively low compared to previous studies, aligning with a lower expected fall rate in our population [7,33]. Accordingly, our low fall incidence is consistent with our methods.

Several reasons might explain why SMT was associated with a reduction in fall risk, yet not associated with a significant difference in limb fracture risk. Given that only about 10% of falls in older individuals lead to extremity fractures [33], the comparable incidence of fractures between cohorts suggests non-fall-related causes, such as motor vehicle collisions or insufficiency fractures (i.e., stress fractures often related to poor bone density).

While our study cannot directly examine the mechanisms behind the observed reduction in falls among those receiving SMT, we can speculate on potential factors. SMT has been shown to reduce pain in individuals with spinal disorders, and pain is known to increase fall risk in older adults [8-10]. Additionally, SMT has been found to improve markers of sensorimotor function and multisensory integration in older adults [14], which may enhance stability and coordination, helping to prevent falls. These factors may collectively contribute to the decreased fall risk observed in those receiving SMT, although further research is needed to explore these mechanisms.

Our secondary limb fracture outcome was limited as we focused on falls in our selection criteria and propensity-matching model to avoid overmatching and sample reduction, thereby omitting potential risk factors for fracture (e.g., glucocorticoids, body weight). Furthermore, fractures may result from several external factors unrelated to falls, such as motor vehicle collisions or other traumas. Limited validity of this outcome is further suggested by the curvilinear increase in incidence during the first week of follow-up, suggestive of confounding by indication. While limb fracture incidence was similar between cohorts, caution is warranted in interpreting this outcome, as our study was primarily tailored to examine falls.

Our findings hold potential clinical significance when considering the high morbidity and cost associated with falls in older individuals [2]. Falls can contribute to pain and accelerate disability progression in this population [8]. To further investigate the clinical implications of our observed reduction in falls associated with SMT, a follow-up study could explore markers of care utilization, pain levels, and associated costs. Such an investigation would allow for a more comprehensive understanding of the potential impact of SMT on older individuals.

Future studies should also consider examining fall-related injuries as a primary outcome, requiring both a fall and injury to ensure clinical relevance when comparing SMT and non-SMT groups. Such studies could explore morbidity and mortality related to fractures and other injuries as secondary outcomes, providing insight into the implications of falls in older individuals. Specifically, a prospective, randomized design, such as a pragmatic trial, would be ideal for assessing the effectiveness of SMT on fall-related injuries and their health consequences. Alternatively, establishing a patient registry for SMT in older adults could facilitate data collection and help attain an adequate sample size.

Strengths and limitations

Study strengths include methods to minimize bias such as requiring preceding and follow-up medical visits, selection of patients from academic medical centers, matching strategy, integrative author team, a priori protocol, and large sample size. In addition, we only required one SMT visit, akin to an intention-to-treat approach, to avoid biases that might arise by only selecting patients who may have had a positive response to care. Finally, adequate data quality metrics, propensity matching diagnostics, and balanced negative control outcomes provide evidence that our methods to minimize observational biases were successful.

Several limitations should be noted. As an observational study, we cannot determine whether SMT was causal in reducing falls. Patients or healthcare providers may have under-reported falls [23]; however, the similarity in negative control outcomes suggests a minimal between-cohort difference regarding reporting/documentation bias. There may be residual confounding related to muscle strength, socioeconomic status, education level, spinal pain-related disability or pain intensity [9,10], time of year [32], and balance measures, which were poorly represented in the dataset. Data in patients’ records could be inaccurate. It is possible some patients with a recent unreported fall were inadvertently included. We could not determine the specific reasons, mechanisms, or circumstances for each fall, as the aggregate nature of the dataset prevents access to detailed patient-level information. It was not feasible to examine our query accuracy against chart review.

Patients in the SMT and non-SMT cohorts may have received education or advice for fall prevention, which was not readily identifiable in the dataset. Similarly, chiropractic treatment regimens may have included non-SMT interventions that were not readily identifiable, such as soft tissue and exercise therapies. We could not further characterize the specific type or technique of SMT applied with respect to the magnitude of force used, patient position, or other features, aside from having been administered by a chiropractor. We were unable to reliably determine the proportion of individual spinal pain diagnoses (e.g., neck vs. low back pain, radiculopathy) due to data aggregation. Findings may not be generalizable to those at a high fall risk who were excluded in the present study. Study results may only generalize to the US, considering potential differences in the management of spinal pain, use of SMT, or fall risk prevalence elsewhere.

We did not require a fall-related injury or emergency visit in our primary outcome. Such a combined outcome could have been more specific and/or clinically relevant, yet it was not feasible given the TriNetX software constraints. A follow-up study could examine injurious falls, yet would require greater patient-level detail and a larger sample size, considering only about 17% of falls result in a documented fall-related injury [33]. A randomized controlled trial could help overcome biases present in our observational design yet may require multicenter participation to attain a sufficient sample size.

## Conclusions

Our findings, representing the largest study to date on SMT and falls, suggest a potential reduction in fall risk after SMT in older adults with spinal pain over 13 months of follow-up compared to matched controls. However, clinical interpretation should remain cautious due to the observational study design, limited ability to explore fall-related injuries, and the lack of significant difference in limb fracture risk. Further research, including randomized controlled trials, is needed to assess the broader clinical implications of SMT, particularly with regard to injurious falls, care utilization, pain, and costs.

## Figures and Tables

**Figure 3:**
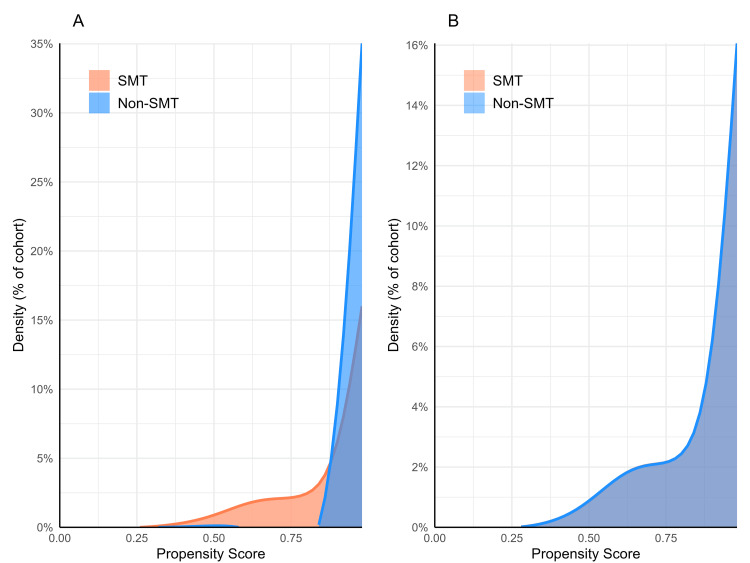
Propensity score density graph Propensity scores before (A) and after (B) matching. The SMT cohort is shown in orange while the non-SMT cohort is shown in blue. After matching, there is a close overlap in the propensity score densities, suggestive of adequate covariate balance. SMT: spinal manipulative therapy

## Appendices

Appendix 1: spinal disorder definition

**Table 3:** Spinal disorder definition *International Classification of Diseases 10th Edition (ICD-10)

Diagnosis code*	Definition
M50-M54	Other dorsopathies
M50	Cervical disc disorders
M51	Thoracic, thoracolumbar, and lumbosacral intervertebral disc disorders
M53	Other and unspecified dorsopathies, not elsewhere classified
M54	Dorsalgia (e.g., cervicalgia, pain in the thoracic spine, low back pain)

Appendix 2: medical examination

**Table 4:** Medical examination CPT: Current Procedural Terminology, SNOMED: Systemized Nomenclature of Medicine

Procedure code	Definition
444971000124105 (SNOMED)	Annual wellness visit
99387 (CPT)	Initial comprehensive medicine evaluation and management, 65 years and older
99397 (CPT)	Periodic comprehensive medicine evaluation and management, 65 years and older
G0438 (CPT)	Annual wellness visit, initial
G0439 (CPT)	Annual wellness visit, subsequent
Z00.0 (CPT)	Encounter for general adult medical examination

Appendix 3: exclusion codes

**Table 5:** Exclusion codes CPT: Current Procedural Terminology, ICD-10: International Classification of Disease 10th Edition, ICD-10-PCS: International Classification of Diseases 10th Edition procedure coding system, TNX: TriNetX custom code

Variable	Definition	Excluded (days)
Exclusions for both cohorts		
F07Z4ZZ (ICD-10-PCS)	Wheelchair mobility treatment	-365 to 0
F01-F09 (ICD-10)	Mental disorders due to known physiological conditions (including dementia)	-365 to 0
Z99.3 (ICD-10)	Dependence on wheelchair	-365 to 0
97542 (CPT)	Wheelchair management (e.g., assessment, fitting, training), each 15 minutes	-365 to 0
W00-W19 (ICD-10)	Slipping, tripping, stumbling, and falls	-91 to 0
TNX	Visit: emergency	Day 0 only
TNX	Visit: inpatient	Day 0 only
1013729 (CPT)	Critical care services	Day 0 only
Z74.01 (ICD-10)	Bed confinement status	Day 0 only
1003143 (CPT)	Surgery	Day 0 only
Exclusions for the non-SMT cohort only		
98940, 98941, 98942 (CPT)	Chiropractic spinal manipulation	0 to 395
97140 (CPT)	Manual therapy (includes mobilization/manipulation techniques)	0 to 395
1013558	Osteopathic manipulative treatment procedures	0 to 395

Appendix 4: propensity-matched variables

**Table 6:** Propensity-matched variables ATC: Anatomical Therapeutic Chemical classification, ICD-10: International Classification of Disease 10th Edition, VANDF: Veterans Health Administration National Drug File

Variable	Description
Demographics	Age, sex
Comorbidities (ICD-10)	
F30-F39	Mood (affective) disorders, including depression
F40.248	Other situational type phobia (i.e., fear of falling)
H25-H28	Disorders of the lens (includes cataracts)
H30-H36	Disorders of choroid and retina
H40-H42	Glaucoma
H53-H54	Visual disturbances and blindness
H81	Disorders of vestibular function (includes vertigo)
H90	Conductive and sensorineural hearing loss
H91	Other and unspecified hearing loss
I30-I15A	Other forms of heart disease (includes arrhythmia)
I50	Heart failure
I95	Hypotension
M15-M19	Osteoarthritis
M48.0	Spinal stenosis
M80-M85	Disorders of bone density and structure
N30-N39	Other diseases of the urinary system (includes urinary incontinence)
R26	Abnormalities of gait and mobility
R42	Dizziness
Z00-Z13	Persons encountering health services for examinations (includes screenings)
Z40-Z53	Encounters for other specific health care (includes postprocedural aftercare)
Z74	Problems related to care provider dependency (includes reduced mobility)
Z55-Z65	Adverse socioeconomic and psychosocial circumstances
Z91.81	History of falling
Medications	
AH000 (VANDF)	Antihistamines
C07 (ATC)	Beta blocking agents
CN101 (VANDF)	Opioid analgesics
CN300 (VANDF)	Sedatives/hypnotics (includes benzodiazepines)
CN600 (VANDF)	Antidepressants
CV702 (VANDF)	Loop diuretics
N02BF (ATC)	Gabapentinoids
N05A (ATC)	Antipsychotics
Visits/procedures	
97116 (CPT)	Gait training
1018495 (CPT)	Intraocular lens procedures (includes cataract removal)

Appendix 5: negative control outcomes

**Table 7:** Negative control outcomes CPT: Current Procedural Terminology, SNOMED: Systemized Nomenclature of Medicine, TNX: TriNetX custom code, HCPCS: Healthcare Common Procedure Coding System, ICD-10-PCS: International Classification of Disease 10th Edition Procedure Coding System

Variable	Description
Colonoscopy	
0DJD8ZZ (ICD-10-PCS)	Inspection of the lower gastrointestinal tract, via natural or artificial opening (endoscopic)
1022231 (CPT)	Colonoscopy, flexible
73761001 (SNOMED)	Colonoscopy
444783004 (SNOMED)	Screening colonoscopy
G0104 (HCPCS)	Colorectal cancer screening; flexible sigmoidoscopy
G0105 (HCPCS)	Colorectal cancer screening; colonoscopy on individual at high risk
G0121 (HCPCS)	Colorectal cancer screening; colonoscopy on individual not meeting criteria for high risk
Nicotine/tobacco screening	
64234-8 (LOINC)	Current smoker
72116-2 (LOINC)	Tobacco smoking status
8664-5 (LOINC)	Cigarettes smoked total (pack per year) - reported
88031-0 (LOINC)	Smokeless tobacco status
F17 (ICD-10)	Nicotine dependence
G9902 (HCPCS)	Patient screened for tobacco use and identified as a tobacco user
G9903 (HCPCS)	Patient screened for tobacco use and identified as a tobacco non-user
Z72.0 (ICD-10)	Tobacco use
Z87.891 (ICD-10)	Personal history of nicotine dependence
Diabetes screening	
9037^ TNX^	Hemoglobin A1c in blood
9025^ TNX^	Hemoglobin A1c measurement
43396009 (SNOMED)	Glucose (mass/volume) in serum, plasma, or blood
36048009 (SNOMED)	Glucose measurement
Vital signs	
9073^TNX^	Respiratory rate
9074^TNX^	Heart rate
9076^TNX^	Body temperature
9081^TNX^	Body weight
9083^TNX^	Body mass index
9085^TNX^	Blood pressure, systolic
9086^TNX^	Blood pressure, diastolic

 Appendix 6: propensity score density graph
